# A Classic Herbal Formula Guizhi Fuling Wan for Menopausal Hot Flushes: From Experimental Findings to Clinical Applications

**DOI:** 10.3390/biomedicines7030060

**Published:** 2019-08-18

**Authors:** Mingdi Li, Andrew Hung, Hong Li, Angela Wei Hong Yang

**Affiliations:** 1 School of Health and Biomedical Sciences, RMIT University, Bundoora, VIC 3083, Australia; 2 School of Science, RMIT University, Melbourne, VIC 3001, Australia

**Keywords:** Cinnamon twig and Poria cocos pill, menopause, literature review, herbal medicine, women’s health, natural products, integrative medicine

## Abstract

A classic herbal formula Guizhi Fuling Wan (GFW) has been used for managing menopausal hot flushes (MHFs), but the evidence across different study types has not been systematically summarized. This project investigated the clinical effects, phytochemistry, pharmacodynamics, and potential mechanisms of actions of GFW on the causative target proteins potentially driving MHFs. Twenty English and Chinese databases were searched for relevant clinical and experimental studies. A total of 12,988 studies were identified, of which 46 were included. Seven clinical studies demonstrated GFW had no statistically significant changes in the frequency and severity of MHFs; however, it could improve peripheral blood flow in the fingertips, jaw, and toes. Thirty-five studies on phytochemistry identified 169 chemical compounds of GFW. Four experimental studies revealed GFW’s therapeutic effects (e.g., normalize calcitonin gene-related peptide (CGRP) level) and potential target protein/cytokine (estrogen receptor beta (ESR2) with genetic variation, CGRP receptor, and interleukin-8) on MHFs. Therapeutic effects across different study types were inconsistent, possibly due to the dose difference and genotype variety of ESR2 in the human population. Further clinical and experimental studies, as well as biochemical investigation on the mechanisms of actions of GFW, are recommended.

## 1. Introduction

A menopausal hot flush (MHF) is a sudden and transient onset of erythema and warmth or burning sensation on the face and skin of the neck which appears during the transition to (pre-menopausal and peri-menopausal), and through, menopause (menopausal and post-menopausal) [1,2]. The onset commonly lasts for seconds to five minutes, but its persistence duration is unpredictable [3]. MHFs significantly impact the quality of life of both the sufferers and their families [3]. Its mechanism has not been fully investigated. The sudden reduction in estrogen level, the narrowed central thermoneutral zone, changes of the certain neurotransmitter synthesis (e.g., noradrenaline and serotonin), and peripheral vascular reactivity are linked to MHF onset [4,5,6,7,8,9]. Menopausal hormone therapy is the most popular and effective MHF management [10,11]. However, adverse effects, such as nausea, dizziness, dry mouth, and contraindication, with hormone-dependent diseases (e.g., breast cancer) exist [12]. Thus, more than half of all middle-aged menopausal women have taken non-hormonal therapies [13], such as Chinese herbal medicine (CHM).

Guizhi Fuling Wan (GFW, also known as Keishibukuryogan) is a classic Chinese herbal formula that has been used for MHFs in modern clinical practice [14,15]. In ancient China, there was no specific terminology used for MHFs. One Classic book, Ying Er Lun (Treatise on Infants), advocated that GFW could be used to treat flushed complexion in females of which the description is similar to MHF symptoms. As recorded in Jin Gui Yao Lue Fang Lun (Synopsis of Prescriptions of the Golden Chamber) [16], GFW consists of five herbal ingredients, including Gui Zhi (Cinnamomi Ramulus), Shao Yao (Paeoniae Radix Alba or Paeoniae Radix Rubra), Mu Dan Pi (Moutan Cortex), Tao Ren (Persicae Semen), and Fu Ling (Poria) [16]. The 2015 edition of the Chinese Pharmacopeia indicates that Bai Shao (Paeoniae Radix Alba) is used in tablet and capsule form GFW, whereas Chi Shao (Paeoniae Radix Rubra) is used in honey pill form [17]. In 2006, a GFW product (capsule) was approved by the US Food and Drug Administration to enter Phase II clinical trials [18]. Recently, more experimental studies have been conducted to investigate the pharmacodynamic effects of GFW [19,20,21]. However, GFW’s application and mechanisms of actions on MHFs have not been systematically reviewed. This paper investigated the therapeutic effects of GFW on the management of MHFs from clinical and experimental perspectives.

## 2. Clinical Application of GFW on MHFs

The formula name and its synonyms were searched as keywords in 20 electronic databases (Cochrane Library, PubMed, EMBASE, AMED, CINAHL, Informit, Science Direct, LILACS, ProQuest, Wiley Online Library, PsycINFO, IndMED, AcuBriefs, Ingenta, KoreaMed, ERIC, CNKI, CQVIP, Wanfang Data, and sinoMed) for English or Chinese language papers from the earliest records available in each database, up to April 2019. The reference lists of articles with relevant topics were manually screened to identify potentially relevant studies. After removal of duplicates, the titles and abstracts of identified potential studies were screened. Full-texts were obtained for eligibility assessment against the selection criteria. All types of clinical studies (including randomized controlled trials (RCTs), non-randomized parallel controlled studies, and case series) were considered for inclusion as long as they recruited patients with MHFs or used MHFs as one of the outcome measures. To ensure comparability of the intervention, only studies using GFW in non-modified forms (e.g., decoction, pills, granules, and capsules with acceptable variations of Bai Shao/Chi Shao/Shao Yao) were considered. For studies involving control groups, only those that compared GFW with placebo, no treatment, or conventional medicine were considered. Studies utilizing any co-interventions were excluded.

A predesigned form was used to record extracted data, including study design, setting, sample size, the age, and diagnosis of the participants, intervention, duration, and outcome measures (including frequency and severity of MHFs, level of hormones/cytokines, and peripheral blood flow). Corresponding authors of potential articles were contacted by email, Research Gate (www.researchgate.net), and LinkedIn (www.linkedin.com) for missing data. Replies with unpublished data were received from corresponding authors of two papers [22,23]. The quality of included studies was analyzed according to study types. For RCTs, the quality was assessed using Cochrane’s risk of bias assessment tool [24] with focus on the following domains: random sequence generation (selection bias), allocation concealment (selection bias), blinding of participants and personnel (performance bias), blinding of outcome assessment (detection bias), incomplete outcome data (attrition bias), and selective reporting (reporting bias). Quality was categorized as “Low risk”, “Unclear risk”, and “High risk” of bias. The quality of parallel controlled studies was evaluated by ROBINS-I (“Risk Of Bias In Non-randomised Studies - of Interventions”) [25], and the assessed domains included confounding, participant selection, intervention classification, deviations from intended interventions, missing data, outcome measurement, and reported results. Quality was classified as “Low risk”, “Moderate risk”, “Serious risk”, and “Critical risk” of bias. The quality of case series was assessed in four domains according to the Instrument for Evaluating the Quality of case series in CHM, including study aims and design (two questions), descriptions of treatment protocol (three questions), descriptions of methods and therapeutic/side-effects (two questions), and conduct of the study (six questions) [26]. A score of “1” was given when ‘Yes’ was applied to one item. Any article with a total score ≥ 7 was considered to have good quality.

**Figure 1:**
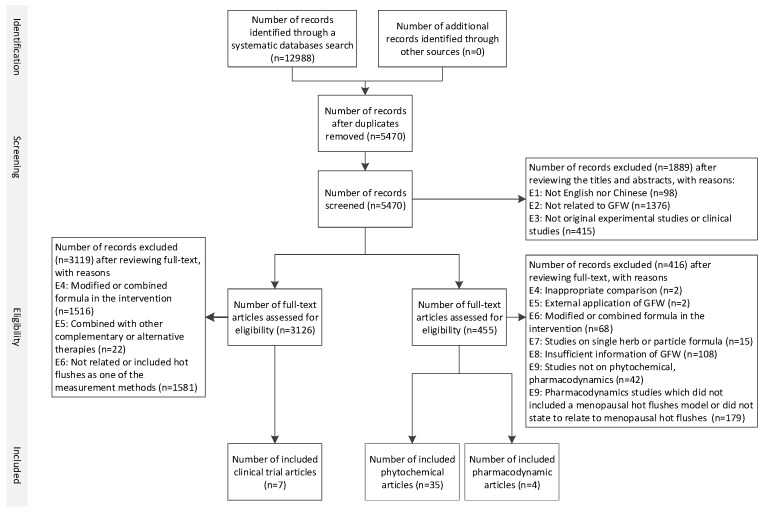
Flowchart of study selection procedures of Guizhi Fuling Wan on menopausal hot flushes.

A total of 12988 records were identified, and seven of them met the inclusion criteria. Figure 1 illustrates the selection process of the included studies. Two RCTs [14,15], one parallel controlled study [27], and two case series [28,29] were included in this review. Another two studies [22,23] were claimed as controlled trials; however, two intervention groups were incomparable. Thus, the treatment groups of these two studies were considered as case series studies, and only data from GFW groups were extracted for analyses in this review. The characteristics of the included studies are summarized in Table 1.

### 2.1. RCTs

One RCT [15] reported that both low dosage (7.5 g/day) and high dosage (12.5 g/day) GFW could significantly reduce MHF frequency and severity when comparing between before and after the 12-week intervention. When compared to placebo, there was no statistical significance. However, the reduction may be dose-dependent, as the results from the high dosage group demonstrated a greater reduction, which provided a direction for future research on the relationship between GFW dosage and clinical effects. Another RCT [14] reported that the peripheral blood flow in postmenopausal females with MHFs in the GFW group (*n* = 67) was significantly decreased compared to the menopausal hormone therapy group (*n* = 64) under the jaw (mean difference (MD) –3.56, 95% confidence interval (CI) –5.17 to –1.95) and in the middle fingertip (MD –7.10, 95% CI –10.99 to –3.21) at the end of one-month treatment. Opposite effects on the blood flow in the third toe were observed in two groups: GFW increased the blood flow in the toe, whereas menopausal hormone therapy caused a decrease.

### 2.2. Parallel Controlled Study

The parallel controlled study [27] reported positive results, concluding that the severity of MHFs in 73.7% of the participants improved from severe to mild or moderate after six-month GFW intake. It was significantly higher than the control group. No significant difference in estradiol (E_2_) and follicle-stimulating hormone (FSH) levels was observed between groups. Results of serum cytokine level indicated that serum monocyte chemotactic protein-1 (MCP-1) level in women treated with keishibukuryogan decreased significantly (–16.3%) compared to the no treatment group (3.8%). Further analysis of the GFW responder group (*n* = 28) indicated that concentrations of serum interleukin (IL)-8 and macrophage inflammatory protein (MIP)-1β were reduced significantly, whereas those in the non-responders were increased dramatically. Sub analysis results on menopausal transition status showed that GFW decreased the IL-8 and MIP-1β levels in perimenopausal GFW responders and MCP-1 in postmenopausal GFW responders.

### 2.3. Case Series

One case series study [29] revealed that the improvement of MHF severity was related to genetic variation of the polymorphic dinucleotide (CA) repeat of the estrogen receptor beta (ESR2) gene on chromosome 14, which include three types: two short alleles (SS), two long alleles (LL), and a short and long allele (SL) [30,31]. MHF severity in participants with LL genotype improved significantly after the intervention. FSH level changes also depended on genotype: FSH levels decreased in participants with SS genotype but increased in participants with SL or LL genotypes. E_2_ level decreased in all genotype groups. Another case series [28] reported that the plasma calcitonin gene-related peptide (CGRP) level in eight post-menopausal participants significantly decreased after the four-week GFW intervention when compared to baseline data (MD –2.88, 95% Cl –4.07 to –1.69). CGRP is a potent vasodilator neuropeptide, and it is hypothesized to be a specific vasodilation neuropeptide of MHFs, which significantly rose in concentration during MHF onset [32]. However, GFW’s effects on changes of frequency before and after intervention in three case series had no statistically significant difference (standardized mean difference (SMD) –1.15, 95% CI –2.49 to 0.20) [22,23,28].

## 3. Experimental Studies on Phytochemistry and Pharmacodynamics of GFW on MHFs

We searched the 20 English and Chinese databases, detailed in Section 2, to identify experimental studies that have investigated the chemical compounds of GFW. Studies were excluded if they did not provide the following details: (1) plants with valid plant specimen voucher numbers, ingredients and ratios for raw herbs (decoction), or (2) company names and batch/lot numbers for purchased patent products (pills, granules, or capsules).

For pharmacodynamics, the same set of 20 databases was searched to identify experimental studies that have investigated GFW on MHFs. To be eligible to be included, the studies must provide the details as specified in (1) and (2) as above. Also, experiments were required to have (3) utilized cell or animal models related to MHFs; (4) compared GFW’s effects with the blank model control group (i.e., no treatment); (5) evaluated the pharmacodynamic effects on MHFs. Studies involving any co-interventions were excluded.

The structures of chemical compounds were drawn using BIOVIA Draw 2019 [33] or Avogadro [34]/Discovery Studio Visualizer 2019 [35]. Molecular weight was calculated by Lenntech Molecular Weight Calculator (https://www.lenntech.com/calculators/molecular/molecular-weight-calculator.htm). The quality of in vivo studies was assessed based on 20 domains specified in the Animal Research: Reporting of In Vivo Experiments (ARRIVE) guidelines [36].

As a result, 35 studies on phytochemistry, and four studies on pharmacodynamics met the inclusion criteria. Figure 1 illustrates the selection process of the included studies.

### 3.1. Phytochemistry of GFW

**Figure 2:**
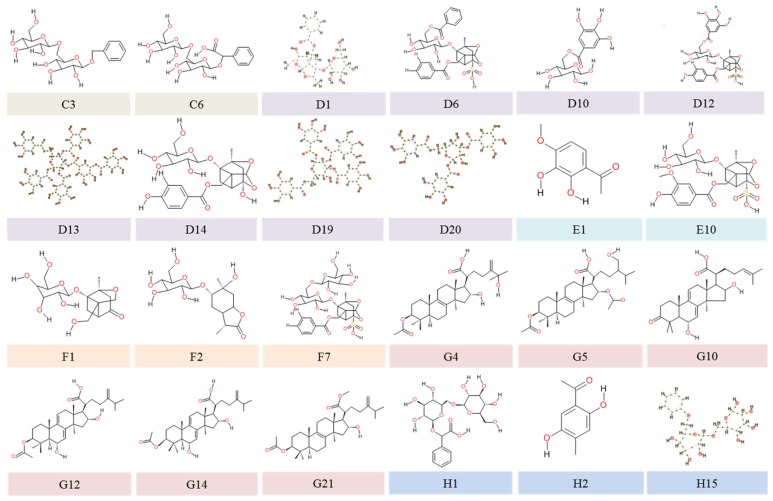
Molecular structures of the 24 chemical constituents of Guizhi Fuling Wan. Note: C3: Benzol-β-gentiobioside, C6: Mandelic acid gentiobioside/isomer, D1: 4′-O-galloypaeoniflorin, D6: Benzoylpaeoniflorin sulfate, D10: Galloyglucose/isomer, D12: Galloylpaeoniflorin sulfonate, D13: Hexagalloyl glucose, D14: Isoxypaeoniflora, D19: Tetragalloy glucose, D20: Trigalloy glucose, E1: 3-hydroxypaeonol, E10: Mudanpioside E sulfonate, F1: 1-O-β-d-glucopyranosyl-paeonisuffrone, F2: 6-O-β-d-glucopyranosyl lactinolide, F7: Isomaltopaeoniflorin sulfonate, G4: 25-Hydroxypachymic acid, G5: 31-Hydroxyl-16-O-acetylpachymic acid, G10: 3-oxo-6,16α-dihydroxy-lanosta-7,9(11),24(31)-trien-21-oic acid, G12: 3β,16α-Dihydroxy-lanosta-7,9(11),24-trien-21-oic acid, G14: 6α-Hydroxydehydropachymic acid, G21: Pachymic acid methyl ester, H1: (2R)-[(6-O-β-d-glucopyranosyl-β-d-glucopyranosyl) oxy] (phenyl) ethanoic acid, H2: 2,5-dihydroxy-4-methylacetophenone, H15: Benzyl-β-d-glucopyranosyl-(1→6)-β-d-glucopyranoside.

The included studies on phytochemicals from GFW reported 169 compounds: four from Cinnamomi Ramulus (A1 and B1–B3), 13 from Persicae Semen (A1 and C1–C12), 41 from Moutan Cortex (A1, D1–D20, and E1–E20), 31 from Paeoniae Radix (A1, D1–D20, and F1–F10), 31 from Poria (A1 and G1–G30), 44 without clearly defined resources (H1–H44), and 29 inorganic elements. Table 2 outlines the characteristics of chemical constituents isolated from GFW. The molecular structures of 144 phytochemicals could be identified with their corresponding PubChem CIDs (compound identification number)/SIDs (substance identification number), whereas 25 constituents were not available in the PubChem database. Among those 25 chemical components, structures of 23 phytochemicals (C3, C6, D1, D6, D10, D12, D13, D14, D19, D20, E1, E10, F1, F2, F7, G4, G5, G12, G14, G21, H1, H2, H15) were provided in the included studies. One compound structure (G10) was available in a reference paper (as cited in Table 2), whereas one (G11) could not be found in any known data source. Details of the 24 molecular structures are presented in Figure 2.

The latest standards for different GFW forms established by high-performance liquid chromatography (HPLC) to guarantee the quality of GFW were cinnamic acid (B2) ≥72 µg and paeonol (E14) ≥6 mg per pill in honey pill form; paeonol (E14) ≥1.8 mg, paeoniflorin (F9) ≥3 mg, and amygdalin (C2) ≥1.5 mg per tablet in tablet form; paeonol (E14) ≥1.8 mg, paeoniflorin (F9) ≥3 mg, and amygdalin (C2) ≥ 0.9 mg per capsule in capsule form [17].

### 3.2. Pharmacodynamic Effects of GFW on MHFs

**Figure 3:**
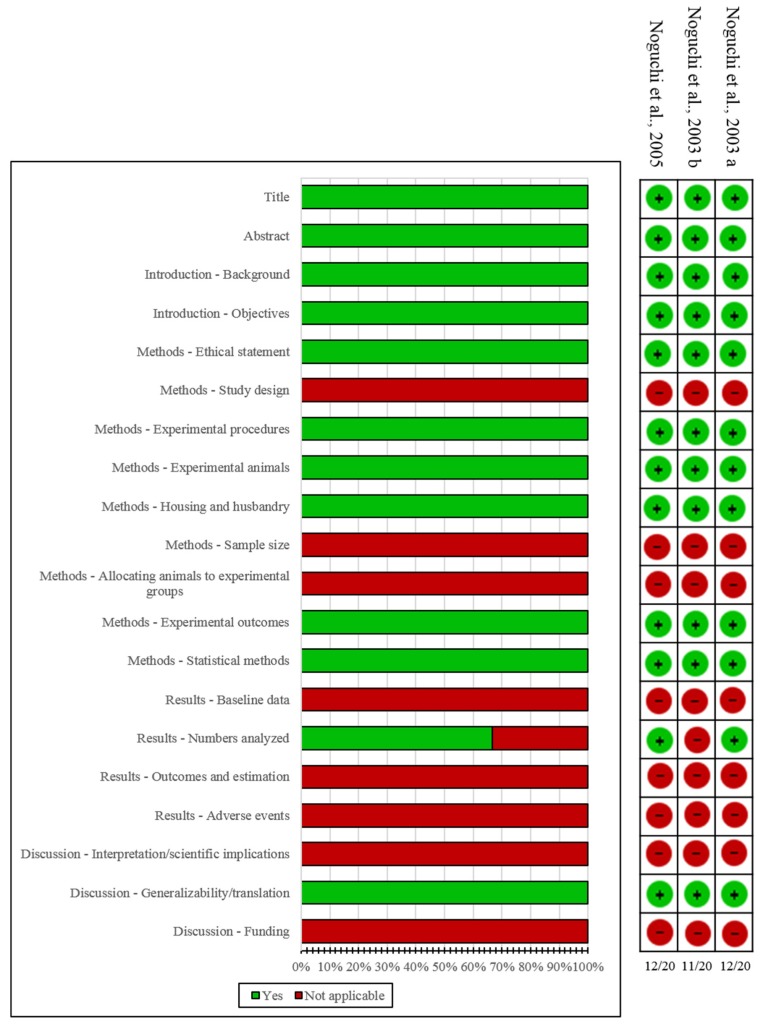
Quality assessment of included in vivo studies according to ARRIVE (Animal Research: Reporting of In Vivo Experiments) guideline.

Characteristics and results of included in vivo studies are summarized in Table 3. The results from the quality assessment of included in vivo experimental studies are presented in Figure 3. The results suggested that GFW had a similar action to E_2_ on managing skin temperature by restoring the plasma level of CGRP: it increased the ovariectomy-induced CGRP reduction [66,67] and reduced CGRP-induced elevation of skin temperature in GnRH (gonadotropin-releasing hormone) analog-treated rats [67,68]. A dose-dependent skin temperature change was detected, showing that a significant inhibition effect on elevated skin temperature was observed at a dose of 1000 mg/kg [67,68]. However, GFW had no significant effects on CGRP concentrations and CGRP mRNA levels in the dorsal root ganglia [66], which suggested minor effects on CGRP synthesis in an ovariectomized rat model. GFW had no significant effects on CGRP concentrations in the spinal cord [66], which suggested that it may have an inconsequential influence on CGRP’s effects on the central nervous system in the ovariectomized rat model.

One in vitro experimental study [69] utilized an ESR-dependent cell proliferation bioassay and an ESR-dependent reporter assay to investigate the potential estrogenic activity of GFW and its metabolites. The results demonstrated that GFW showed no estrogenic activity and low ER β-dependent estrogenic activity before or after metabolization with a concentration at 100 μg/mL. Findings from two in vivo studies concluded that E_2_ could significantly reduce the levels of pituitary LH (luteinizing hormone) and FSH, as well as the weights of uterus and ovaries, whereas GFW did not influence those factors [67,68]. Thus, GFW did not confer estrogen activity on plasma and might exert pharmacodynamic effects against MHFs via other pathways.

## 4. Translation from Experimental Studies to Clinical Practice

### 4.1. Therapeutic Effects

**Figure 4:**
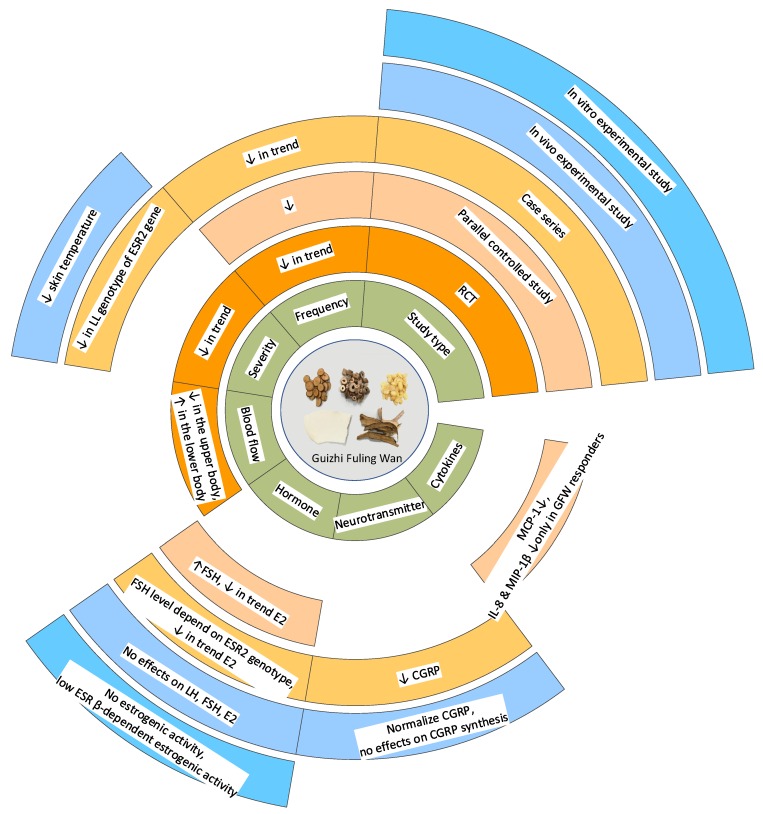
Therapeutic effects of Guizhi Fuling Wan on menopausal hot flushes across different types of studies. Notes: CGRP: calcitonin gene-related peptide; E_2_: estradiol; ESR: estrogen receptor; FSH: follicle-stimulating hormone; 1IL: interleukin; LH: luteinizing hormone; LL: two long alleles; MIP: macrophage inflammatory protein; MCP: serum monocyte chemotactic protein; RCT: randomized controlled trial.

A well-designed RCT is considered as the gold standard when evaluating the effects of an intervention [70]. However, its limitations have triggered extensive discussion as RCTs may be unnecessary, inappropriate, inadequate, or even impossible in the clinic, and methodological problems can cause bias in the results [71,72,73]. Besides, CHM involves a holistic therapeutic approach. However, most of the published RCTs on CHM were designed to only investigate the therapeutic aspects (i.e., Chinese medicine intervention + western medicine diagnosis) instead of the holistic therapeutic approach (i.e., Chinese medicine intervention + Chinese medicine diagnosis) [74]. The importance of integration of various study designs was highlighted to accommodate the evaluation of various research questions and for the outcome of interest [75,76]. Therefore, the therapeutic effects of a range of study designs were systematically reviewed (Figure 4).

As shown in Figure 4, the study types and outcome measures varied across all the included studies. Most outcomes were measured in different study designs. GFW demonstrated overall no statistically significant changes in the frequency and severity of MHFs between before and after treatment and no statistically significant differences between GFW and control groups. However, dose-dependent skin temperature changes were detected in both clinical [15] and experimental studies [69,70]. The effective dose suggested in the in vivo study was 1000 mg/kg [69,70], which was four times the dose in the RCT (12.5 g/day) when the participant weighed in at 50 kg [15]. Thus, more dose variation should be considered in further RCTs. The influence of GFW on FSH and E_2_ was unclear based on the included studies due to the limitation of study design. Results of experimental studies indicated that GFW had no effects on LH, FSH, and E_2_ in Sprague-Dawley rats and no estrogenic effects on the rat liver S9 fraction. However, case series studies pointed out that GFW might target a specific human ESR2 beta genotype. GFW was found to affect CGRP level in both case series and in vivo studies [28,66,67,69]. GFW had similar effects to E_2_ on normalizing CGRP level but without affecting its synthesis in the dorsal root ganglia. Only peripheral blood flow and cytokines were reported in one study design, which should be further investigated in various studies.

### 4.2. Potential Multi-Targeting Actions

Three potential target proteins/cytokine could be proposed based on findings from the above-mentioned clinical and experimental studies: estrogen beta receptor, IL-8, and CGRP receptor.

There are significant associations between the ESR2 CA dinucleotide repeat length and other menopausal-related symptoms. For example, low bone mineral density has been reported previously in pre- and post-menopausal women [31,77,78]. This genetic variation was characterized by Tsukamoto and co-workers in 1998 [79]. Recently, ESR2 CA dinucleotide repeat length was reported to be closely related to clinical effects of GFW on MHFs and that it had better performance on participants with the LL genotype [29]. Women with the SS genotype had significantly higher values of circulation estrone concentration (highest) when compared to those harboring the LL genotype (lowest) after adjustment for other confounding factors, including age, hormone replacement status, and circulating levels of sex hormone-binding globulin [31]. Thus, genetic variation should be considered as an essential factor of using GFW for MHFs to achieve precision treatment. The prevalence of the SS and SL genotypes was approximately 47.79% (108/226) and 22.56% (51/226), respectively [31]. The sum of these two genotypes was much higher than that of the LL genotype (29.65%, 67/226). Thus, patients without targeting genotype might contribute to the negative results in the RCT. It is suggested that future research focus on the three genotypes of ESR2 for precision medicine.

IL-8, also known as CXCL8, is a cytokine potentially involved in thermoregulation and was detected to be higher in premenopausal and menopausal women with MHFs than in those without MHFs [80,81]. GFW could significantly reduce concentrations of serum IL-8, whereas those in the non-responders were increased dramatically [27]. Thus, GFW might selectively influence the synthesis of IL-8. However, the underlying mechanism of this selective effect in different MHF sufferers is not fully understood.

CGRP is a widely expressed sensory neuropeptide, which plays a major role in modulating metabolism, inflammatory response, and blood pressure, as well as contributing to nerve development and function [82,83,84,85,86]. In the sympathetic nervous system, CGRP is believed to be the specific vasodilator responsible for MHFs [4,84]. Recently, Liang et al. [86] have determined the structure of the human CGRP receptor in complex with CGRP, with the Gs-protein heterotrimer at 3.3 Å global resolution, which makes it possible for future investigation of the potential herb-target relationship between GFW and MHF-related target protein.

### 4.3. Safety

Inorganic elements that may have vital biological activities in the human body were traced in GFW, including calcium, sodium, magnesium with a dose over 300 µg/g, followed by iron, manganese, and strontium with a dose over 10 µg/g [65]. The dosage of toxic inorganic elements detected in the included studies included beryllium, arsenic, cadmium, antimony, mercury, thallium, and bismuth, which were under 0.03 µg/g and were lower than the maximum limit level listed in the Chinese Pharmacopoeia [65]. GFW is believed to be a safe herbal product as only a small number of minor gastrointestinal symptoms were reported as adverse effects: two studies reported adverse events, including diarrhea (*n* = 15) [15] and abnormal feeling in the gastrointestinal tract (*n* = 2) [27]. They were the main causes leading to drop out. Additionally, an in vivo study on Sprague-Dawley rats indicating that GFW was not carcinogenic after a continuous 24-month intervention [87]. Results from another in vitro experiment [68] on estrogen-dependent human breast cancer (MCF-7) cells showed that GFW at concentrations of 10.6–10.4 mg/mL did not activate the proliferation of MCF-7 cell, which suggested that GFW did not exhibit estrogen activity. Therefore, GFW might be a potential therapeutic option for MHFs in women who are undergoing breast cancer treatment.

### 4.4. Implications for Future Research

In total, 169 compounds from GFW and three potential target proteins/cytokine (estrogen beta receptor, IL-8, and CGRP receptor) were identified based on findings from the above-mentioned clinical and experimental studies. However, at this stage, a clear understanding of their interactions could not be obtained based on current literature. The bioactivities of the phytochemicals and their mechanisms of actions on MHF were not fully investigated. Although quality control is improving during the last two decades, evidenced by the increased quantitative index from one in the 2005 edition to two or three in the 2015 edition of the Chinese Pharmacopeia [17], not all the herbal ingredients have a quantitative index. The clinical effects of CHM are often regarded as the result of the multi-targeting interaction of various phytochemical compounds. Thus, more research on the mechanism of actions of CHM on a condition at the molecular level (ligand-target interaction) and the therapeutic dose of the bioactive compounds are warranted.

## 5. Conclusions

This study identified 169 compounds from GFW pills, tablets, and capsules. Therapeutic effects, including frequency and severity of MHFs, peripheral blood flow, hormones, neurotransmitter, and cytokines, were not consistent across different study types. ESR2 with genetic variation, CGRP receptor, and IL-8 were identified to be related to the mechanisms of actions of GFW on MHFs. The differences in therapeutic effects could be potentially due to dose variations among clinical and experimental studies and ESR2 gene differences in rats and homo species. Further clinical and experimental studies, as well as biochemical investigation on the mechanisms of actions of GFW, are warranted. It is recommended that the dose-dependent effects and ESR2 expression at the participant recruitment stage are considered in future investigation.

## Figures and Tables

**Table 1:** Characteristics and quality assessment of included studies of Guizhi Fuling Wan for menopausal hot flushes.

Study Name	Setting (Country)	Sample Size; Age	Diagnosis	Interventions	Duration	Outcomes Involved in this Review	Quality Assessment
**RCT**							
Plotnikoff et al. 2011 [15]	University (US)	178; 45–58	Post-menopausal	T1: GFW at 7.5 g/day (*n* = 62); T2: GFW at 12.5 g/day (*n* = 57); C: Placebo (*n* = 59)	12 weeks (12 weeks duration + 1-week placebo run-in period)	Frequency and severity of MHFs	L, L, L, L, L, H
Ushiroyama et al. 2005 [14]	College (Japan)	140; T: 53.1 ± 6.5; C: 53.5 ± 6.2	Post-menopausal	T: GFW 7.5 g/day (*n* = 70); C: oral HT (Premarin R 0.625 mg/day + Provera R: 2.5 mg/day) (*n* = 70)	1 month	Blood flow in peripheral tissue	U, U, H, U, H, L
**Parallel Controlled Study**							
Yasui et al. 2011 [27]	Outpatient clinic of a university hospital (Japan)	77; T: 52 (39.3–58.7); C: 51 (43.4–57)	Pre-menopausal, peri-menopausal, post-menopausal, and bilateral oophorectomized	T: GFW 7.5 g/day (*n* = 38); C: No treatment (*n* = 39)	6 months	The severity of MHFs, FSH, and E_2_ levels, IL-8, MCP-1, MIP-1β	L, L, L, L, M, S, L
**Case Series**							
Chen and Shiraki 2003 [28]	Clinic (Japan)	8; 53.59 ± 0.58	Post-menopausal	GFW at 7.5 g/day (*n* = 8)	4 weeks	Frequency of MHFs, plasma CGRP level	High-quality (score = 8)
Namiki et al. 2014 [29]	Hospital and clinic of university (Japan)	39; 49.5 ± 4.69 (for 34 patients)	Post-menopausal (*n* = 18), ‘not yet menopausal’ (*n* = 21)	GFW at 2.5 g, 3 times/day (*n* = 39)	12 weeks	Severity of MHFs, FSH, and E_2_ levels	High-quality (score = 10)
Terauchi et al. 2010 [22]	Hospital and clinic of university (Japan)	16; 51.1 ± 2.4	Peri- and post-menopausal	Education + GFW 7.5 g/day (*n* = 16)	144 ± 58 days	Frequency of MHFs	High-quality (score = 8)
Terauchi et al. 2011 [23]	Hospital and clinic of university (Japan)	30; 50.5 ± 5.2	Peri- and post-menopausal	Education + GFW 7.5 g/day (*n* = 30)	182 ± 76 days	Frequency of MHFs	High-quality (score = 7)

Notes: C: control group; CGRP: calcitonin gene-related peptide; E_2_: estradiol; FSH: follicle-stimulating hormone; GFW: Guizhi Fuling Wan; H: high risk; MHFs: menopausal hot flushes; L: low risk; M: moderate risk; RCT: randomized controlled trial; S: serious risk; T: treatment group; U: unclear risk. Quality of RCTs was assessed Cochrane’s risk of bias assessment tool [24], quality of parallel controlled studies was evaluated by ROBINS-I (“Risk Of Bias In Non-randomised Studies - of Interventions”) [25], and case series were assessed according to the Instrument for Evaluating the Quality of case series in Chinese herbal medicine [26].

**Table 2:** Chemical compounds from Guizhi Fuling Wan.

ID	List of Chemical Compounds	Form of GFW	Molecular Formula	Molecular Weight (g/mol)	PubChem CID/SID	Methods
**A**	**All ingredients (*n* = 1)**					
A1	Sucrose/isomer (isomer only in Paeoniae radix)	Capsule	C12H22O11	342.297	5988	LC-MS/UPLC-ESI-Q-TOF-MS [37]
**B**	**Cinnamomi Ramulus (*n* = 3)**					
B1	Cinnamaldehyde	Capsule, pill	C9H8O	132.162	637511	HPLC [38,39], SPE-HPLC [40], UPLC [41]
B2	Cinnamic acid	Capsule, pill	C9H8O2	148.161	444539	HPLC [38,39], multiple chromatographic methods [42], HPLC-ESI-Q-TOF/MS [43], DGLC [44], UPLC [41], HPLC-MS/MS [45]
B3	Protocatechuic acid	Capsule	C7H6O4	154.121	72	LC-MS/UPLC-ESI-Q-TOF-MS [37], multiple chromatographic methods [42]
**C**	**Persicae Semen (*n* = 12)**					
C1	A-Linolenic acid		C18H30O2	278.436	5280934	HPLC [46]
C2	Amygdalin	Capsule, tablet	C20H27NO11	457.432	656516	LC-MS/UPLC-ESI-Q-TOF-MS [37], HPLC [47,48,49,50,51], HPLC-ESI-Q-TOF/MS [43], DGLC [44], UPLC [41], HPLC-MS/MS [45]
C3	Benzol-β-gentiobioside	Capsule	C19H28O11	432.42	NF (Figure 2)	LC-MS/UPLC-ESI-Q-TOF-MS [37]
C4	Betulinic acid	Capsule	C30H48O3	456.711	64971	LC-MS/UPLC-ESI-Q-TOF-MS [37]
C5	Linoleic acid	Capsule	C18H32O2	280.452	5280450	LC-MS/UPLC-ESI-Q-TOF-MS [34], HPLC [46]
C6	Mandelic acid gentiobioside/isomer	Capsule	C20H28O13	476.43	NF (Figure 2)	LC-MS/UPLC-ESI-Q-TOF-MS [37]
C7	Mandelic acid-β-d-glucopyranoside	Capsule	C14H18O8	314.29	73229629	LC-MS/UPLC-ESI-Q-TOF-MS [37]
C8	Oleic acid	Capsule	C18H34O2	282.468	445639	LC-MS/UPLC-ESI-Q-TOF-MS [37], HPLC [46]
C9	Palmitic acid	Capsule	C16H32O2	256.43	985	LC-MS/UPLC-ESI-Q-TOF-MS [37]
C10	Prunasin	Capsule	C14H17NO6	295.291	119033	LC-MS/UPLC-ESI-Q-TOF-MS [37]
C11	Stearic acid	Capsule	C18H36O2	284.484	5281	LC-MS/UPLC-ESI-Q-TOF-MS [37]
C12	Trametenolic acid	Capsule	C30H48O3	456.711	12309443	LC-MS/UPLC-ESI-Q-TOF-MS [37]
**D**	**Paeoniae Radix + Moutan Cortex (*n* = 20)**					
D1	4′-O-galloypaeoniflorin	Capsule	C30H32O15	632.56	NF (Figure 2)	LC-MS/UPLC-ESI-Q-TOF-MS [37]
D2	4-hydroxybenzoic acid	Capsule	C7H6O3	138.122	135	LC-MS/UPLC-ESI-Q-TOF-MS [37], multiple chromatographic methods [42]
D3	4-O-galloylalbiflorin	Capsule	C30H32O15	632.571	135397096	LC-MS/UPLC-ESI-Q-TOF-MS [37]
D4	Albiflorin	Capsule	C23H28O11	480.466	51346141	LC-MS/UPLC-ESI-Q-TOF-MS [37], RP-HPLC [52], chromatography on silica gel, Sephdex LH-20 columns and prep-HPLC-NMR [53], HPLC-ESI-Q-TOF/MS [43], HPLC-MS/MS [45]
D5	Benzoylpaeoniflorin/isomer	Capsule	C30H32O12	584.574	21631106	LC-MS/UPLC-ESI-Q-TOF-MS [37], chromatography on silica gel, Sephdex LH-20 columns and prep-HPLC-NMR [53], HPLC-ESI-Q-TOF/MS [43]
D6	Benzoylpaeoniflorin sulfate	Capsule	C30H32O14S	648.63	NF (Figure 2)	LC-MS/UPLC-ESI-Q-TOF-MS [37]
D7	Desbenzoylalbiflorin/ isomer	Capsule	C16H24O10	376.358	71452333	LC-MS/UPLC-ESI-Q-TOF-MS [37]
D8	Digalloylglucose	Capsule	C20H20O14	484.366	129628549	LC-MS/UPLC-ESI-Q-TOF-MS [37]
D9	Gallic acid	Capsule	C7H6O5	170.12	370	LC-MS/UPLC-ESI-Q-TOF-MS [37], RP-HPLC [52], HPLC-ESI-Q-TOF/MS [43], UPLC [41], HPLC-MS/MS [45]
D10	Galloyglucose/isomer	Capsule	C13H16O10	332.26	NF (Figure 2)	LC-MS/UPLC-ESI-Q-TOF-MS [37]
D11	Galloyl paeoniflorin	Capsule	C30H32O15	632.571	494717	LC-MS/UPLC-ESI-Q-TOF-MS [37], chromatography on silica gel and polydextran gel columns and prep-HPLC-NMR/MS [54], HPLC-ESI-QTOF/MS [43]
D12	Galloylpaeoniflorin sulfonate	Capsule	C30H32O17S	696.63	NF (Figure 2)	LC-MS/UPLC-ESI-Q-TOF-MS [37]
D13	Hexagalloyl glucose	Capsule	C48H36O30	1092.78	NF (Figure 2)	LC-MS/UPLC-ESI-Q-TOF-MS [37]
D14	Isoxypaeoniflora	Capsule	C23H28O12	496.46	NF (Figure 2)	LC-MS/UPLC-ESI-Q-TOF-MS [37]
D15	Methyl gallate	Capsule	C8H8O5	184.147	7428	LC-MS/UPLC-ESI-Q-TOF-MS [37]
D16	Methyl vanillate	Capsule	C9H10O4	182.175	19844	LC-MS/UPLC-ESI-Q-TOF-MS [37]
D17	Oxypaeoniflorin	Capsule	C23H28O12	496.465	21631105	LC-MS/UPLC-ESI-Q-TOF-MS [37], chromatography on silica gel, Sephdex LH-20 columns and prep-HPLC-NMR [53], chromatography on silica gel, Sephdex LH-20 columns, D-101 macroporous resin column, and reverse RP-18 column and HPLC-NMR/MS [55]
D18	Pentagalloylglucose	Capsule	C41H32O26	940.681	65238	LC-MS/UPLC-ESI-Q-TOF-MS [37], HPLC-ESI-Q-TOF/MS [43]
D19	Tetragalloy glucose	Capsule	C34H28O22	788.57	NF (Figure 2)	LC-MS/UPLC-ESI-Q-TOF-MS [37]
D20	Trigalloy glucose	Capsule	C27H24O18	636.47	NF (Figure 2)	LC-MS/UPLC-ESI-Q-TOF-MS [37]
**E**	**Moutan Cortex (*n* = 20)**					
E1	3-hydroxypaeonol	Capsule	C9H10O4	182.17	NF (Figure 2)	LC-MS/UPLC-ESI-Q-TOF-MS [37]
E2	Apiopaeonoside	Capsule	C20H28O12	460.432	127509	LC-MS/UPLC-ESI-Q-TOF-MS [37]
E3	Benzoyloxypaeoniflorin	Capsule	C30H32O13	600.573	21631107	LC-MS/UPLC-ESI-Q-TOF-MS [37], HPLC-ESI-Q-TOF/MS [43]
E4	Galloyloxypaeoniflorin	Capsule	C30H32O16	648.56	3036133	LC-MS/UPLC-ESI-Q-TOF-MS [37]
E5	Mudanpioside A		C31H34O13	614.6	21631101	Chromatography on silica gel and polydextran gel columns and prep-HPLC-NMR/MS [54], HPLC-ESI-Q-TOF/MS [43]
E6	Mudanpioside B	Capsule	C31H34O14	630.599	21631102	LC-MS/UPLC-ESI-Q-TOF-MS [37]
E7	Mudanpioside C	Capsule	C30H32O13	600.573	21631098	LC-MS/UPLC-ESI-Q-TOF-MS [37], chromatography on silica gel and polydextran gel columns and prep-HPLC-NMR/MS [54], HPLC-ESI-QTOF/MS [43]
E8	Mudanpioside D	Capsule	C24H30O12	510.492	21631103	LC-MS/UPLC-ESI-Q-TOF-MS [37]
E9	Mudanpioside E	Capsule	C24H30O13	526.491	21631104	LC-MS/UPLC-ESI-Q-TOF-MS [37]
E10	Mudanpioside E sulfonate	Capsule	C24H30O14S	574.55	NF (Figure 2)	LC-MS/UPLC-ESI-Q-TOF-MS [37]
E11	Mudanpioside F	Capsule	C16H24O8	344.36	21631108	LC-MS/UPLC-ESI-Q-TOF-MS [37], chromatography on silica gel, Sephdex LH-20 columns, D-101 macroporous resin column, and reverse RP-18 column and HPLC-NMR/MS [55]
E12	Mudanpioside H/ isomer	Capsule	C30H32O14	616.572	71457654	LC-MS/UPLC-ESI-Q-TOF-MS [37]
E13	Mudanpioside J	Capsule	C31H34O14	630.599	21593828	LC-MS/UPLC-ESI-Q-TOF-MS [37]
E14	Paeonol	Capsule, pill, tablet	C9H10O3	166.176	11092	LC-MS/UPLC-ESI-Q-TOF-MS [37], HPLC [38,39,47,50,51], gas chromatography [56], RP-HPLC [57,58], SPE-HPLC [40], HPLC-ESI-QTOF/MS [43], DGLC [44], UPLC [41], HPLC-MS/MS [45]
E15	Paeonolide	Capsule	C20H28O12	460.432	442923	LC-MS/UPLC-ESI-Q-TOF-MS [37]
E16	Paeonoside	Capsule	C15H20O8	328.317	53384347	LC-MS/UPLC-ESI-Q-TOF-MS [37]
E17	Suffruticoside A	Capsule	C27H32O16	612.537	9986231	LC-MS/UPLC-ESI-Q-TOF-MS [37]
E18	Suffruticoside B	Capsule	C27H32O16	612.537	10258205	LC-MS/UPLC-ESI-Q-TOF-MS [37]
E19	Suffruticoside C	Capsule	C27H32O16	612.537	10258206	LC-MS/UPLC-ESI-Q-TOF-MS [37]
E20	Suffruticoside D	Capsule	C27H32O16	612.537	5321547	LC-MS/UPLC-ESI-Q-TOF-MS [37]
**F**	**Paeoniae Radix (*n* = 10)**					
F1	1-O-β-d-glucopyranosyl-paeonisuffrone	Capsule	C16H24O9	360.36	NF (Figure 2)	LC-MS/UPLC-ESI-Q-TOF-MS [37]
F2	6-O-β-d-glucopyranosyl lactinolide	Capsule	C16H26O9	362.37	NF (Figure 2)	LC-MS/UPLC-ESI-Q-TOF-MS [37]
F3	Albiflorin R1	Capsule	C23H28O11	480.466	5317181 (CID), 50163461(SID)	LC-MS/UPLC-ESI-Q-TOF-MS [37], chromatography on silica gel, Sephdex LH-20 columns and prep-HPLC-NMR [53]
F4	Digallic acid	Capsule	C14H10O9	322.225	341	LC-MS/UPLC-ESI-Q-TOF-MS [37]
F5	Ethyl gallate	Capsule	C9H10O5	198.174	13250	LC-MS/UPLC-ESI-Q-TOF-MS [37], multiple chromatographic methods [42]
F6	Galloylsucrose/isomer	Capsule	C19H26O15	494.402	129629059	LC-MS/UPLC-ESI-Q-TOF-MS [37]
F7	Isomaltopaeoniflorin sulfonate	Capsule	C29H38O18S	706.66	NF (Figure 2)	LC-MS/UPLC-ESI-Q-TOF-MS [37]
F8	Oxypaeoniflorin sulfonate	Capsule	C23H28O14S	560.523	71455848	LC-MS/UPLC-ESI-Q-TOF-MS [37]
F9	Paeoniflorin	Capsule, pill, tablet	C23H28O11	480.466	442534	LC-MS/UPLC-ESI-Q-TOF-MS [37], HPLC [47,50,51,59,60], RP-HPLC [52], chromatography on silica gel, Sephdex LH-20 columns and prep-HPLC-NMR [53], HPLC-ESI-QTOF/MS [43], DGLC [44], UPLC [41], HPLC-MS/MS [45]
F10	Paeoniflorin sulfonate	Capsule	C23H28O14S	560.523	101382399	LC-MS/UPLC-ESI-Q-TOF-MS [37], HPLC-ESI-QTOF/MS [43]
**G**	**Poria (*n* = 30)**					
G1	16α-Hydroxytrametenolic acid	Capsule	C30H48O4	472.71	132285301	LC-MS/UPLC-ESI-Q-TOF-MS [37], UPLC/Q-TOF-MS [61]
G2	16α-Hydroxydehydrotrametenolic acid	Capsule	C30H46O4	470.694	10743008	UPLC/Q-TOF-MS [61]
G3	2,3,6-Trimethylbenzoic acid	Capsule	C10H12O2	164.204	17314	LC-MS/UPLC-ESI-Q-TOF-MS [37]
G4	25-Hydroxypachymic acid	Capsule	C33H52O6	544.76	NF (Figure 2)	LC-MS/UPLC-ESI-Q-TOF-MS [37]
G5	31-Hydroxyl-16-O-acetylpachymic acid	Capsule	C38H52O5	588.81	NF (Figure 2)	LC-MS/UPLC-ESI-Q-TOF-MS [37]
G6	3-epidehydropachymic acid	Capsule	C33H50O5	526.758	15226716	Chromatography on silica gel, Sephdex LH-20 columns, D-101 macroporous resin column, and reverse RP-18 column and HPLC-NMR/MS [55], HPLC [62], UPLC/Q-TOF-MS [61]
G7	3-Epidehydrotumulosic acid	Capsule	C31H48O4	484.721	10005581	LC-MS/UPLC-ESI-Q-TOF-MS [37], chromatography on silica gel, Sephdex LH-20 columns, D-101 macroporous resin column, and reverse RP-18 column and HPLC-NMR/MS [55], UPLC/Q-TOF-MS [61]
G8	3-O-acetyl-16α hydroxytrametenolic acid	Capsule	C32H50O5	514.747	9958136	LC-MS/UPLC-ESI-Q-TOF-MS [37], UPLC/Q-TOF-MS [61]
G9	3-O-acetyl-16α-hydroxydehydrotrametenolic acid	Capsule	C32H48O5	512.731	15226714	LC-MS/UPLC-ESI-Q-TOF-MS [37], UPLC/Q-TOF-MS [61]
G10	3-oxo-6,16α-dihydroxy-lanosta-7,9(11),24(31)-trien-21-oic acid	Capsule	C30H44O5	484.67	NF (Figure 2) (structure found in [63])	UPLC/Q-TOF-MS [61]
G11	3-oxo-6,16α-dihydroxy-lanosta-8,24-diene-21-oic acid	Capsule	NF	NF	NF (structure not available)	UPLC/Q-TOF-MS [61]
G12	3β,16α-Dihydroxy-lanosta-7,9(11),24-trien-21-oic acid	Capsule	C30H46O4	470.68	NF (Figure 2)	LC-MS/UPLC-ESI-Q-TOF-MS [37]
G13	3β-O-p-Hydroxybenzoyl-dehydro tumulosic acid	Capsule	C38H52O6	604.828	5318155	LC-MS/UPLC-ESI-Q-TOF-MS [37]
G14	6α -Hydroxydehydropachymic acid	Capsule	C33H50O6	542.74	NF (Figure 2)	LC-MS/UPLC-ESI-Q-TOF-MS [37]
G15	Dehydroeburicoic acid	Capsule	C31H48O3	468.722	15250826	LC-MS/UPLC-ESI-Q-TOF-MS [37], UPLC-MS/MS [64], UPLC/Q-TOF-MS [61]
G16	Dehydropachymic acid	Capsule	C33H50O5	526.758	15226717	LC-MS/UPLC-ESI-Q-TOF-MS [37], UPLC-MS/MS [64], HPLC [62], UPLC/Q-TOF-MS [61]
G17	Dehydrotrametenolic acid	Capsule	C32H48O3	480.733	129539661	LC-MS/UPLC-ESI-Q-TOF-MS [37], UPLC-MS/MS [64], UPLC/Q-TOF-MS [61]
G18	Dehydrotumulosic acid	Capsule	C31H48O4	484.721	15225964	LC-MS/UPLC-ESI-Q-TOF-MS [37], UPLC-MS/MS [64], chromatography on silica gel, Sephdex LH-20 columns, D-101 macroporous resin column, and reverse RP-18 column and HPLC-NMR/MS [55], HPLC [62], UPLC/Q-TOF-MS [61]
G19	Eburicoic acid	Capsule	C31H50O3	470.738	73402	LC-MS/UPLC-ESI-Q-TOF-MS [37], UPLC/Q-TOF-MS [61]
G20	Pachymic acid	Capsule	C33H52O5	528.774	5484385	LC-MS/UPLC-ESI-Q-TOF-MS [37], UPLC-MS/MS [64], HPLC-MS/MS [45], UPLC/Q-TOF-MS [61]
G21	Pachymic acid methyl ester	Capsule	C34H54O5	542.79	NF (Figure 2)	LC-MS/UPLC-ESI-Q-TOF-MS [37]
G22	Polyporenic acid C	Capsule	C31H46O4	482.705	9805290	LC-MS/UPLC-ESI-Q-TOF-MS [37], UPLC-MS/MS [64], chromatography on silica gel, Sephdex LH-20 columns, D-101 macroporous resin column, and reverse RP-18 column and HPLC-NMR/MS [55], HPLC [62], UPLC/Q-TOF-MS [61]
G23	Poricoic acid A	Capsule	C31H46O5	498.704	5471851	LC-MS/UPLC-ESI-Q-TOF-MS [37]
G24	Poricoic acid AE	Capsule	C33H50O5	526.758	102480392	LC-MS/UPLC-ESI-Q-TOF-MS [37]
G25	Poricoic acid AM	Capsule	C32H48O5	512.731	46882717	LC-MS/UPLC-ESI-Q-TOF-MS [37]
G26	Poricoic acid B	Capsule	C30H44O5	484.677	5471852	LC-MS/UPLC-ESI-Q-TOF-MS [37]
G27	Poricoic acid BM	Capsule	C31H46O5	498.704	15225967	LC-MS/UPLC-ESI-Q-TOF-MS [37]
G28	Poricoic acid C	Capsule	C31H46O4	482.705	16757534	LC-MS/UPLC-ESI-Q-TOF-MS [37]
G29	Poricoic acid DM	Capsule	C32H48O6	528.73	44424830	LC-MS/UPLC-ESI-Q-TOF-MS [37]
G30	Tumulosic acid	Capsule	C31H50O4	486.737	12314446	LC-MS/UPLC-ESI-Q-TOF-MS [37], UPLC/Q-TOF-MS [61]
**H**	**Unclarified (*n* = 44)**					
H1	(2R)-[(6-O-β-d-glucopyranosyl-β-d-glucopyranosyl) oxy] (phenyl) ethanoic acid	Capsule	C20H28O13	475. 145	NF (Figure 2)	HPLC-ESI-Q-TOF/MS [43]
H2	2,5-dihydroxy-4-methylacetophenone	Capsule	C9H10O3	166.174	NF (Figure 2)	Multiple chromatographic methods [42]
H3	4-o-methylbenzoylpaeoniflorin	Capsule	C31H34O12	598.601	46883517	Chromatography on silica gel, Sephdex LH-20 columns and prep-HPLC-NMR [53]
H4	4-o-methylgalloylpaeoniflorin	Capsule	C31H34O15	646.598	46883518	Chromatography on silica gel, Sephdex LH-20 columns and prep-HPLC-NMR [53]
H5	Adenosine	Capsule	C10H13N5O4	267.245	60961	Chromatography on silica gel, Sephdex LH-20 columns, D-101 macroporous resin column, and reverse RP-18 column and HPLC-NMR/MS [55]
H6	Affinoside	Capsule	C20H28O12	460.432	11972427	Multiple chromatographic methods [42]
H7	Alanine	Capsule	C3H7NO2	89.094	5950	Chromatography on silica gel, Sephdex LH-20 columns, D-101 macroporous resin column, and reverse RP-18 column and HPLC-NMR/MS [55]
H8	A-Amyrin acetate	Capsule	C32H52O2	468.766	92842	Chromatography on silica gel and polydextran gel columns and prep-HPLC-NMR/MS [54]
H9	A-D-Glucose	Capsule	C6H12O6	180.156	79025	Chromatography on silica gel, Sephdex LH-20 columns, D-101 macroporous resin column, and reverse RP-18 column and HPLC-NMR/MS [56]
H10	Apigenin	Capsule	C15H10O5	270.24	5280443	Chromatography on silica gel and polydextran gel columns and prep-HPLC-NMR/MS [54]
H11	Arginine	Capsule	C6H14N4O2	174.204	6322	Chromatography on silica gel, Sephdex LH-20 columns, D-101 macroporous resin column, and reverse RP-18 column and HPLC-NMR/MS [56]
H12	Astragalin	Capsule	C21H20O11	448.38	5282102	Chromatography on silica gel and polydextran gel columns and prep-HPLC-NMR/MS [54]
H13	Benzoic acid	Capsule	C7H6O2	122.123	243	HPLC-ESI-QTOF/MS [43], UPLC [41]
H14	Benzoylpaeoniflorin	Capsule	C30H32O12	584.574	21631106	HPLC-ESI-QTOF/MS [43], UPLC [41]
H15	Benzyl-β-D-glucopyranosyl-(1→6)-β-D-glucopyranoside	Capsule	C18H26O10	402.39	NF (Figure 2)	Multiple chromatographic methods [42]
H16	Β-Amyrin acetate	Capsule	C32H52O2	468.766	92156	Chromatography on silica gel and polydextran gel columns and prep-HPLC-NMR/MS [54]
H17	Caffeic acid	Capsule	C9H8O4	180.159	689043	Chromatography on silica gel, Sephdex LH-20 columns, D-101 macroporous resin column, and reverse RP-18 column and HPLC-NMR/MS [55]
H18	Campesterol	Capsule	C28H48O	400.691	173183	Chromatography on silica gel and polydextran gel columns and prep-HPLC-NMR/MS [54]
H19	Catechin	Capsule	C15H14O6	290.271	9064	Chromatography on silica gel and polydextran gel columns and prep-HPLC-NMR/MS [54]
H20	Cinnamyl alcohol	Capsule	C9H10O	134.178	5315892	Chromatography on silica gel and polydextran gel columns and prep-HPLC-NMR/MS [54]
H21	Coumarin	Capsule	C9H6O2	146.145	323	Chromatography on silica gel and polydextran gel columns and prep-HPLC-NMR/MS [54]
H22	Epicatechin	Capsule	C15H14O6	290.271	72276	Chromatography on silica gel and polydextran gel columns and prep-HPLC-NMR/MS [54]
H23	Ergosta-4, 6, 8 (14), 22-tetraen-3-one	Capsule	C28H40O	392.627	6441416	Chromatography on silica gel and polydextran gel columns and prep-HPLC-NMR/MS [54]
H24	Ergosterol	Capsule	C28H44O	396.659	444679	Chromatography on silica gel and polydextran gel columns and prep-HPLC-NMR/MS [54]
H25	Galactitol	Capsule	C6H14O6	182.172	11850	Chromatography on silica gel, Sephdex LH-20 columns, D-101 macroporous resin column, and reverse RP-18 column and HPLC-NMR/MS [55]
H26	Guanosine	Capsule	C10H13N5O5	283.244	6802	Chromatography on silica gel, Sephdex LH-20 columns, D-101 macroporous resin column, and reverse RP-18 column and HPLC-NMR/MS [55]
H27	Heneicosanoic acid	Capsule	C21H42O2	326.565	16898	Chromatography on silica gel and polydextran gel columns and prep-HPLC-NMR/MS [54]
H28	Hyperoside	Capsule	C21H20O12	464.379	5281643	Chromatography on silica gel and polydextran gel columns and prep-HPLC-NMR/MS [54]
H29	Isomaltopaeoniflorin	Capsule	C29H38O16	642.607	101001429	Chromatography on silica gel, Sephdex LH-20 columns and prep-HPLC-NMR [53]
H30	Kaemferol	Capsule	C15H10O6	286.239	5280863	Chromatography on silica gel and polydextran gel columns and prep-HPLC-NMR/MS [54]
H31	Leucine	Capsule	C6H13NO2	131.175	6106	Chromatography on silica gel, Sephdex LH-20 columns, D-101 macroporous resin column, and reverse RP-18 column and HPLC-NMR/MS [55]
H32	O-β-D-Gentiobiosyl-d-(−)-mandelamide	Capsule	C20H29NO12	498. 158	181802	HPLC-ESI-QTOF/MS [43]
H33	Paeonidanin A	Capsule	C31H34O12	598.601	44253993	Chromatography on silica gel, Sephdex LH-20 columns and prep-HPLC-NMR [53]
H34	Paeonidanin B	Capsule	C31H34O15	646.598	102417825	Chromatography on silica gel, Sephdex LH-20 columns and prep-HPLC-NMR [53]
H35	Paeoniflorin B	Capsule	C36H42O17	746.715	71452334	Chromatography on silica gel, Sephdex LH-20 columns and prep-HPLC-NMR [53]
H36	P-Coumaric acid	Capsule	C9H8O3	164.16	637542	Multiple chromatographic methods [42]
H37	Proline	Capsule	C5H9NO2	115.132	145742	Chromatography on silica gel, Sephdex LH-20 columns, D-101 macroporous resin column, and reverse RP-18 column and HPLC-NMR/MS [55]
H38	Quercetin	Capsule	C15H10O7	302.238	5280343	Chromatography on silica gel and polydextran gel columns and prep-HPLC-NMR/MS [54]
H39	Syringaresinol	Capsule	C22H26O8	418.442	443023	Chromatography on silica gel and polydextran gel columns and prep-HPLC-NMR/MS [54]
H40	Syringic acid	Capsule	C9H10O5	198.174	10742	Chromatography on silica gel and polydextran gel columns and prep-HPLC-NMR/MS [54], HPLC-ESI-QTOF/MS [43]
H41	Trans-2-Methoxycinnamic acid	Capsule	C10H10O3	178.187	734154	Multiple chromatographic methods [42]
H42	Trehalose	Capsule	C12H22O11	342.297	7427	Chromatography on silica gel, Sephdex LH-20 columns, D-101 macroporous resin column, and reverse RP-18 column and HPLC-NMR/MS [55]
H43	Umbelliferone	Capsule	C9H6O3	162.144	5281426	Chromatography on silica gel and polydextran gel columns and prep-HPLC-NMR/MS [54]
H44	Vanillic acid	Capsule	C8H8O4	168.148	8468	Multiple chromatographic methods [42]
**J**	**Inorganic Elements (*n* = 29)**					
J1	Aluminum	Capsule	Al	26.982	5359268	ICP-MS [65]
J2	Arsenic	Capsule	As	74.922	5359596	ICP-MS [65]
J3	Boron	Capsule	B	10.81	5462311	ICP-MS [65]
J4	Barium	Capsule	Ba	137.327	5355457	ICP-MS [65]
J5	Beryllium	Capsule	Be	9.012	5460467	ICP-MS [65]
J6	Bismuth	Capsule	Bi	208.98	5359367	ICP-MS [65]
J7	Calcium	Capsule	Ca	40.078	5460341	ICP-MS [65]
J8	Cadmium	Capsule	Cd	112.414	23973	ICP-MS [65]
J9	Cobalt	Capsule	Co	58.933	104730	ICP-MS [65]
J10	Chromium	Capsule	Cr	51.996	23976	ICP-MS [65]
J11	Copper	Capsule	Cu	63.546	23978	ICP-MS [65]
J12	Iron	Capsule	Fe	55.845	23925	ICP-MS [65]
J13	Gallium	Capsule	Ga	69.723	5360835	ICP-MS [65]
J14	Mercury	Capsule	Hg	200.592	23931	ICP-MS [65]
J15	Lithium	Capsule	Li	6.94	3028194	ICP-MS [65]
J16	Magnesium	Capsule	Mg	24.305	5462224	ICP-MS [65]
J17	Manganese	Capsule	Mn	54.938	23930	ICP-MS [65]
J18	Molybdenum	Capsule	Mo	95.95	23932	ICP-MS [65]
J19	Sodium	Capsule	Na	22.99	5360545	ICP-MS [65]
J20	Nickel	Capsule	Ni	58.693	935	ICP-MS [65]
J21	Lead	Capsule	Pb	207.2	5352425	ICP-MS [65]
J22	Antimony	Capsule	Sb	121.76	5354495	ICP-MS [65]
J23	Selenium	Capsule	Se	78.971	6326970	ICP-MS [65]
J24	Tin	Capsule	Sn	118.71	5352426	ICP-MS [65]
J25	Strontium	Capsule	Sr	87.62	5359327	ICP-MS [65]
J26	Titanium	Capsule	Ti	47.867	23963	ICP-MS [65]
J27	Thallium	Capsule	Tl	204.38	5359464	ICP-MS [65]
J28	Vanadium	Capsule	V	50.941	23990	ICP-MS [65]
J29	Zinc	Capsule	Zn	65.38	23994	ICP-MS [65]

Notes: CID: compound identification number; DGLC: dual gradient liquid chromatography; ESI: electrospray ionization; ICP: inductively coupled plasma; LC: liquid chromatography; MS: mass spectrometry; NF: Not found; NMR: nuclear magnetic resonance spectroscopy; Q: quadrupole; RP: reverse phase; SID: substance identification number; SPE: solid-phase extraction; TOF: time of flight; UPLC: ultra performance liquid chromatography. Corresponding molecular structures refer to PubChem and Figure 2.

**Table 3:** Characteristics of in vivo studies.

Study ID	Language; Location	Model	Inducer	Age (week)	Weight (g)	Experiments	Sample Size	Interventions (Daily Dose, Administration)	Duration (days)	Included Outcome Measure and Results
Noguchi et al., 2003 a [66]	English, Japan	Sprague-Dawley rats	OVX, capsaicin (1 mg/kg, i.p.) injection	10	200–250	1	32 (8/8/8/8)	1. E_2_ (0.010 mg/kg, s.c.); 2. GFW (1000mg/kg, p.o.); 3. Distilled water (10 mL/kg, p.o.) to OVX rats; 4. Distilled water (10 mL/kg, p.o.) to sham-operated rats.	7	CGRP concentrations in plasma ↑ (restore)
			OVX			2	27 (7/7/7/7)			CGRP concentrations in the spinal cord and dorsal root ganglia (no sig)
						3	20 (5/5/5/5)			CGRP mRNA levels in dorsal root ganglia (no sig)
Noguchi et al., 2003 b [67]	English, Japan	Sprague-Dawley rats	OVX	10	200–250	4	NS	1. GFW (100, 300, or 1000 mg/10 mL per kg, p.o., *n* = 7 or 8 in each group); 2. E_2_ (0.010 mg/mL per kg, s.c., *n* = 7); 3. Distilled water (10 mg/kg, p.o., *n* = 8) to OVX rats; 4. GFW (1000 mg/10 mL per kg, p.o., *n* = 8) to sham-operated rats.	7	CGRP-induced elevation of skin temperature ↓
						5	45 (11/13/12/9)	1. GFW (1000 mg/10 mL per kg, p.o.); 2. E_2_ (0.010 mg/mL per kg, s.c.); 3. Distilled water (10 mL/kg, p.o.) to OVX rats; 4. Distilled water (10 mL/kg, p.o.) to sham-operated rats.		CGRP-induced relaxation of Prostaglandin F2α-induced vasoconstriction in isolated mesenteric vascular beds ↓
						6	32 (8/8/8/8)			125I-CGRP binding in isolated mesenteric arteries ↓
						7	38 (10/9/10/9)			Plasma concentration of CGRP ↑
						8	29 (7/7/8/7)			Plasma concentration of E_2_ (no sig); uterine weight (no sig)
Noguchi et al., 2005 [68]	English, Japan	Sprague-Dawley rats	GnRH analog Leupline (1 mg/kg) injection	9	200–240	9	36 (6/6/5/7/7/5)	1. GFW (100 mg/kg, p.o.); 2. GFW (300 mg/kg, p.o.); 3. GFW (1,000 mg/kg, p.o.); 4. E_2_ (0.010 mg/kg, s.c.); 5. Distilled water (10 mL/kg, p.o.) to injected rats; 6. Distilled water (10 mL/kg, p.o.) to sham-injected rats.	14	CGRP-induced elevation of skin temperature ↓
							52 (13/13/13/13)	1. GFW (1000 mg/kg, p.o.); 2. E_2_ (0.010 mg/kg, s.c.); 3. Distilled water (10 mL/kg, p.o.) to injected rats; 4. Distilled water (10 mL/kg, p.o.) to sham-injected rats.		Plasma concentration of CGRP ↑
							28 (8/7/7/6)			Pituitary LH and FSH, plasma E_2_ and weights of uterus and ovaries (no sig)

Notes: ↑: increased; ↓: decreased; CGRP: calcitonin gene-related peptide; E_2_: estradiol; FSH: follicle-stimulating hormone; GFW: Guizhi Fuling Wan; GnRH: gonadotropin-releasing hormone; i.p.: intraperitoneal injection; LH: luteinizing hormone; NS: not specified; OVX: ovariectomized; p.o.: oral administration; s.c.: subcutaneous injection; sig: significance.
